# The Relationship of Personality Style and Attention Deficit Hyperactivity Disorder in Children

**Published:** 2017-05-15

**Authors:** Stephen P. Amos, Gretchen J. Homan, Natalie Sollo, Carolyn R. Ahlers-Schmidt, Matthew Engel, Patrice Rawlins

**Affiliations:** Department of Pediatrics, University of Kansas School of Medicine-Wichita, KS

**Keywords:** attention deficit hyperactive disorder, Myers Briggs Type Indicator, personality inventory, pediatrics

## Abstract

**Introduction:**

This study was to identify personality correlates of children with a diagnosis of Attention Deficit Hyperactive Disorder (ADHD). The Jungian Personality Type dimensions primarily considered were Sensing/Intuiting and Perceiving/Judging. A Sensing child is likely to be very present-centered. A Perceiving child tends to be curious and resist order and structure.

**Methods:**

Children attending a general pediatric clinic with a diagnosis of ADHD were eligible to participate. Enrolled children were administered the Murphy-Meisgeier Type Indicator for Children. Binomial tests were performed comparing Perceiving and Sensing personality components to accepted population rates.

**Results:**

Participants (n = 117) were predominantly male (78%) with a median age of 10 years. The Sensing trait (72%) was more prevalent than expected, though prevalence for the Perceiving trait (44%) did not differ from population rates.

**Conclusion:**

Personality types occasioned with the diagnosis of ADHD could be useful in establishing/normalizing treatment regimens and approaches to assist these children and their families better.

## Introduction

Attention Deficit/Hyperactivity Disorder (ADHD) is a condition characterized by high levels of hyperactivity/impulsivity and inattention that affects up to 10% of school-age children.^1^ ADHD is associated with chronic functional impairment and increased risk for later psychopathology.^2^,^3^ The specific disorder, as defined by the *Diagnostic and Statistical Manual of Mental Disorders* (DSM-V), includes operational criteria targeting both behaviors and deficits in abilities including inattention and communication/impulsivity.^4^ A search of the literature focused on the relationship of personality characteristics/traits and ADHD revealed a preponderance of research identifying negative aspects associated with the diagnosis, including increased risk of injury, reduced educational achievement, and economic impact.^5^,^6^ There was a paucity of research aimed at identifying positive aspects of the diagnosis or ways in which ADHD symptomatology combines favorably with life’s demands. In addition, most research of ADHD and personality focused on adults and not children.

Once thought to disappear with maturation, longitudinal studies have shown ADHD symptoms generally manifest themselves in early childhood, prior to age 12, and can be present in some form throughout adulthood.^6^–^8^ Depending on informant and diagnostic cutoff points, anywhere from 5 to 75% of adults diagnosed as children show significant levels of impairment into adulthood.^9^ Some have suggested a relationship between disorders of neurocognitive and/or executive function (e.g., ADHD) and subsequent psychopathology (e.g., personality disorders) in adulthood.^6^,^10^ However, others have argued the constructs associated with ADHD may be adaptive and represent a positive adjustment to a disorganized and chaotic world.^11^,^12^

Core symptoms of ADHD may shift in adulthood.^13^ Behaviors such as difficulty maintaining attention and frequent running around shift to affective lability, lack of anger management skills, emotional over-reactivity, and disorganization. However, coupled with this are concomitant spontaneity, creativity, and responsiveness. Many of the traits associated with creative individuals overlap substantially with behavioral descriptions of ADHD, including higher levels of spontaneous idea generation, mind wandering, daydreaming, sensation seeking, energy, and impulsivity.^14^ In addition, persons with diagnosed ADHD may be more likely to convert the exhaustive effects of the disorder into exceptional qualities. Barkley^15^ noted that children with ADHD actually are able to concentrate intently; this is especially true when the endeavor interests them or provides immediate reinforcement and feedback. Those with an ADHD diagnosis activate higher levels of creative thought and achievement than people without the diagnosis.^16^,^17^ This leads to questions concerning what factors contribute to success of those with ADHD and whether they might be functions of personality.

The key constructs of ADHD often appear to be transient. Hyperactivity often declines by adolescence but problems with attention remain.^18^ Impulsivity may transform from acting without thinking into executive function issues including problems in self-reflection, planning, and creating a future orientation that anticipates outcomes. However, this also may give way to fearless negotiation of life circumstances that sometimes leads to surprisingly creative solutions.^19^ Adults with ADHD also reported occasional bursts of activity leading to adaptability, learning to overcome difficulties, and a moderate risk-taking agenda that allows them to disregard obstacles that prevent others from even exploring new possibilities.^17^,^20^

Many studies have looked at ADHD through the lens of pathognomonic indicators, such as the Millon Clinical Multiaxial Indicator or the Minnesota Multiphasic Personality Inventory II.^21^,^22^ ADHD often is associated with depression, anxiety, and lower self-esteem as expressions of increased difficulties at home and in the educational setting.^3^,^8^,^22^,^23^ Fewer studies have sought to identify the positive aspects of ADHD as capable of influencing adaptive functioning in certain situations and as a precursor to success rather than a pathway to failure. For example, adults with ADHD are nearly four times as likely to be entrepreneurs as their counterparts without the disorder.^18^

In response to increasing interest in understanding individual personality differences, Carl Jung’s theory of psychological type has been used to develop tools to identify personality indicators.^24^ The essence of the theory is that perceived random variation in human behavior is orderly and consistent, being due to certain basic differences in the way people prefer to use their perception and judgment.^25^ The Myers-Briggs Type Indicator (MBTI) was the first tool developed to investigate Jung’s ideas and measures preferences of the four polar dimensions: Extraversion/Introversion, Sensing/Intuiting, Thinking/Feeling, and Judging/Perceiving. According to type theory, all eight of these preferences are used by each of us but they are not preferred equally. The Murphy-Meisgeier Type Indicator (MMTIC) was developed in an attempt to expand such investigation into the lives of children. The MMTIC reflects normal and adaptive development without any reflection of pathology.^26^ As each individual grows and develops, predisposed preferences emerge regarding how that person will operate and transact in the world. To date there has been little research looking into the relationship between individual personality type in populations of children with ADHD.

The current version of MMTIC has been constructed carefully and the combined reliability and validity statistics demonstrate it is appropriate for and accurately assesses preferences for grades 2 thru 12.^26^ One particular value of the MMTIC is that it demonstrates clear expectancies of type for the general population. For example, approximately 54% of children would be Judging in their orientation to the world and approximately 46% would be Perceiving. Judging children tend to be planful, organized, orderly, and systematic, whereas Perceiving children tend to be creative, curious, open, flexible, and adaptive, but somewhat scattered in terms of organization. Likewise, expectancies for Sensing and Intuiting would be 57% and 43%, respectively. Sensing children archetypally are present-centered observers who like to do things now, one step at a time, paying attention to details with little regard for the future. Alternatively, Intuiting children tend to look to the future seeking patterns and relationships with a focus on the big picture but often missing details.

The primary research goal was to determine the extent to which an ADHD diagnosis is associated with certain personality preferences. This research explored the possibility that ADHD carries a predisposition to experience the world in certain ways that may complicate the delivery of treatment services and the way in which children with ADHD actually use treatment services. Given the aforementioned descriptions of these personality types, we proposed that Sensing/Perceiving children would not be a natural fit for some educational settings. In addition, their individual preferences may predispose them to be identified as having ADHD. We hypothesized that children with ADHD would be more likely to express the Sensing and Perceiving dimensions on the MMTIC. The Extraversion/Introversion or Thinking/Feeling dimensions were not expected to differ from established population frequencies.

## Methods

Patients between grade levels 2 and 12 presenting to the practicing psychologist at a general pediatrics clinic in Wichita, KS, who previously were diagnosed with ADHD (all types), were asked to participate in this study. Recruitment occurred between May 2011 and March 2015. For this study, ADHD was defined as confirmed diagnosis by a pediatrician and required additional documentation utilizing the Conner’s Behavior Rating Forms, both Parent and Teacher.^27^,^28^

Age, grade level, and gender were collected for all enrolled participants. Each participant was asked to complete the MMTIC. The 43-item assessment tool has documented reliability between .69 and .78 for each of the four scales (Extraversion/Introversion, Sensing/Intuiting, Thinking/Feeling and Judging/Perceiving).^26^ Children completed the instrument using a computerized assessment (Center for Applications of Psychological Type, Inc; www.capt.org). Basic frequencies were calculated for each of the four dimensions as well as the combinations of all four dimensions. Observed frequencies of individual types were compared to expected values taken from the MMTIC Manual.^26^

Given the small sample size and skewed distribution of age and grade level, non-parametric tests were used. Age and gender of respondents for each dimension were compared using Mann-Whitney U and Fisher’s exact tests, respectively. Frequencies of MMTIC preferences were compared to expected values using binomial test of proportion. Analyses were performed using SPSS (IBM SPSS Version 20.0). Significance was defined as p < 0.05. T-test and chi-squared tests were two-tailed. The binomial test of proportion is a one-tailed test. This project was approved by the institutional review board at the Wichita Medical Research and Education Foundation.

## Results

All children with a verified diagnosis of ADHD seen by the psychologist were enrolled (n = 117). Children were mostly male (78%), with a median age of 10 (interquartile range [IQR] 8 – 12), and were in the 4^th^ grade (IQR 3 – 6). The most common 4-type personality indicator was ISFJ (Table 1). Table 2 describes the percent of each personality type who were male and the median age for each type. Age and gender were significantly associated with trait preferences across dimensions (age unassociated with Feeling/Thinking dimension, p = 0.074, all others unassociated, p > 0.2).

When compared to expected averages taken from the MMTIC manual, children in our sample were more likely to exhibit the Sensing preference (72%) than would have been expected (57%; p = 0.001). No differences were detected in the expression of the Perceiving preference (44%) as compared to the expected 46% (p = 0.334).Differences were detected in both the proportion expressing the Introversion preference (58%; p = 0.001) and the Thinking preference (29%; p = 0.005). Respectively, these are compared to expected frequencies of 43% and 41%.

## Discussion

The results of this study affirmed our hypothesis that children with ADHD were more likely to be Sensing on the MMTIC, but did not support that they are more likely to exhibit the Perceiving trait. These results presented an intriguing picture of ADHD and personality type that warrants future research, especially at pediatric clinics where ADHD is a relatively common diagnosis. It would be important to see if children with an ADHD diagnosis are indeed more likely to be Sensing in their personality style. Sensing children may live in the present moment without much thinking or worrying about the future and often like real things that are right now. They prefer going step-by-step in a concrete fashion and principally are not interested in theories or big picture generalizations that are usually part of the instructional field of play in any educational system. These children tend to be pragmatic and practical and if the lesson does not make sense to them they will disregard it because the lesson has no place in their worldview. It could be expected that Sensing children would have trouble with an educational system designed to teach big concepts that have little to no real meaning for the practical world they live. Conversely, Intuiting children tend to be quick in their ability to get the major concepts being taught but often miss the details leading to the larger lesson.

The Judging/Perceiving dimension is equally intriguing. Judging children tend to be organized and systematic, while Perceiving children tend to be more curious and playful in their approach to the outside world, including education. Children with a Judging preference may value getting things done and often enjoy schedules and routines. Judgers tend to be neat, orderly, and like completing their work on time. They frequently cannot consider playing if they have an assignment due. Perceiving children tend to be far more flexible and like to have time open to do whatever they want whenever they want to do it. They may start lots of projects but have difficulty actually getting anything done. The importance of the spontaneous moment can be a powerful enticer for the Perceiving child. This is precisely why we expected children with ADHD to be inclined to be more Perceiving in their orientation; however, the data in our sample did not support this hypothesis.

There were other incidental findings in this research regarding higher than expected expression of Introversion and Feeling. It may be that these preferences grew in response to the impairments associated with ADHD, for example, difficulty forming and maintaining friendships or heightened sensitivity to educational impediments. However, further research is necessary.

This research pointed to the importance of knowing who the patient is, just as much as knowing what the patient has. The utilization of the MMTIC afforded the opportunity to do just that and to tailor approaches to intervention to fit the personal style of the child. It also allowed the opportunity to think more globally with parents about why a child does what they do, not just in terms of ADHD, but also in terms of who they are as people. This process also suggested where effort needs to be placed in terms of educational interventions and in treatment especially regarding cognitive behavioral approaches. For example, interventions that require a long-term investment and delayed gratification might not bear as much fruit as those devised in a playful, present centered way, with a reward that is immediate rather than delayed.

Perhaps, the more important outcome of this research is the consideration of the personality orientation of the child in addition to a focus on the specific ADHD dimensional criteria. We would suggest that adding the MMTIC to standard ADHD assessment techniques such as behavior rating scales and computer generated tests may create a more complete picture of the child we hope to help.

## Figures and Tables

**Table 1:** Distribution of personality types.*

**ISTJ**	**ISFJ**	**INFJ**	**INTJ**
5.1%	20.5%	5.1%	0.9%
**ISTP**	**ISFP**	**INFP**	**INTP**
6%	13.7%	3.4%	3.4%
**ESTP**	**ESFP**	**ENFP**	**ENTP**
2.6%	4.3%	5.1%	5.1%
**ESTJ**	**ESFJ**	**ENFJ**	**ENTJ**
5.1%	14.5%	4.3%	0.9%

* Extraversion/Introversion, Sensing/Intuiting, Thinking/Feeling, and Judging/Perceiving.

**Table 2:** Age and Gender by type

	% Male	Median age
Extroversion	73%	10
Introversion	81%	10

Intuiting	76%	10
Sensing	79%	10

Feeling	76%	10
Thinking	82%	10

Perceiving	75%	10
Judging	80%	10
